# Public perspectives on strays and companion animal management in Malaysia

**DOI:** 10.1186/s12889-023-16276-5

**Published:** 2023-07-26

**Authors:** Syafiq Munir Ismail Munir, Mohd Istajib Mokhtar, Ahmad Firdhaus Arham

**Affiliations:** 1 Department of Science and Technology Studies, Faculty of Science, University of Malaya, Kuala Lumpur, Malaysia; 2 Pusat Pengajian Citra Universiti, Universiti Kebangsaan Malaysia, UKM, Bangi, Selangor Malaysia

**Keywords:** Companion Animal Management, Strays, Animal Overpopulation, Animal Regulation and Implementation, Urban Ecology Sustainability, Malaysia

## Abstract

Strays and companion animal management is a sensitive issue in Malaysia that incites solid and conflicting views. Through structured questionnaires administered to a random sample of 704 respondents, this study explored public opinion on a) causes of the stray animal population, b) the management of the stray animal population, and c) the national strategy on strays and companion Animal Management. The results show that 70.3% of respondents agreed that a lack of public awareness regarding animal care was the major contributor to the stray animal population. In addition, 58.1% of respondents felt that treating and vaccinating animals exposed to zoonotic diseases is a viable approach that could be instituted as a reasonable measure in stray animal population management. Finally, developing animal protection areas through a multi-stakeholder partnership strategy initiative recorded the highest support (48.4%) for intervention planning for stray animal management at a national level. Notably, a significant percentage of public responses were implicitly influenced by demographic variables. These findings provide valuable insights into public opinion regarding stray and companion animal management in Malaysia. These findings could inform the development of future legislation aimed at reducing the unfavorable effects of stray animal populations on humans and the ecology of Malaysia.

**Supplementary Information:**

The online version contains supplementary material available at 10.1186/s12889-023-16276-5.

## Introduction

The current scenario regarding the global stray animal population is overwhelming. The evidence shows that dog populations are estimated to total 900 million [1, 2], 83% of which are unrestrained [3]. The worldwide feral cat population is estimated at least 100 million, including about 60 million in the United States [4]. In Malaysia, cats and dogs are the two most popular terrestrial animals chosen as a pet [5]. The ownership of dogs stood at 402,500 and cats at 795,000 in 2018 [6, 7]. At the same time, recent development shows that Malaysia was one of the top 10 countries where the pet humanization rate had spiked in 2020 from the past five years [8].

Chances for owned pets to be assimilated and diffused into the stray animal population are possible if the owner cannot control the excess number of animals, ineffective containment until the animal freely escapes to the external environment, irresponsible breeding, and improper pet care. All these factors have sped up feral and stray animal populations blooming. While the number of stray dogs and cats in Malaysia is still unknown, it is believed the numbers are tremendous, totaling approximately 6 million and 5 million, respectively, compared to the number of owned pet dogs and cats in the country [9].

Moreover, companion animals such as cats and dogs can have both positive and negative effects on the social community. On one hand, they can provide companionship and emotional support in human-animal interaction contributing to better well-being [10]. However, when their numbers are out of control or overpopulated, they can also pose a threat to public health and safety. They can transmit zoonoses [11, 12], including rabies [13, 14], leishmaniasis [15], toxocariasis [16], giardiasis, and other zoonotic parasites that negatively affect the health and well-being of both humans and animals. Additionally, stray animals create issues pertaining to waste, sanitation and street nuisance. They often suffer from road accidents and injuries [17–19]. Therefore, issues related to strays and companion animal management are part and parcel of various multifaced science and social problems that require direct public understanding when creating a management strategy. Regarding intervention implementation, the Malaysian regulations on dog culling do not necessarily align with the World Organization for Animal Health (OIE) standards on animal welfare. Consequently, it reduces the overall score in the Animal Protection Index (API) 2020 which signifies a reduction in Malaysia’s legislation and policy commitment to protect animals. While the OIE accepts that euthanasia of strays may be required, it is the last resort for animals that cannot be re-homed. Adoption and re-homing should be prioritized according to OIE standards [20].

Limited research in Malaysia regards to stray animal management and measure implementation related to it was identified. Previous research focused on the existence of microbes that potentially causes transmissible zoonotic disease from one animal to another species, including cats, dogs, and humans [21, 22]. Another study recently focused on assessing and comparing stray cats’ health conditions between 4 different localities in Johor and identified the existence of six different stray cat breeds in the State [23].

A previous study by Yong (2015) focuses on addressing the approach and conveying suggestions about managing the problems and issues caused by the overpopulation of stray cats in the Universiti Teknologi Malaysia (UTM) campus [24]. The study was conducted restrictedly within a specific university campus. Thus, it lacks taking phenomena that could contribute to increasing stray numbers, such as limiting pet numbers on-premises and breeding regulation. Another recent study by Dorothy et al. (2019) focuses on public preferences for trap-neuter-release (TNR) and trap-euthanasia (TE) programs for free-roaming dogs in Penang [25]. The survey results suggested that the public is concerned about free-roaming dogs and encouraged authorities to seek improved methods of population control that are humane and acceptable to society. However, the small sample population involved a survey of 157 Penangites. The research needs to explore public opinion on how government can enforce strays’ intervention measures starting from its sources, such as limits on cat numbers, breeding regulations, mandatory identification, and desexing.

According to Walker et al. (2017), they identified variations in New Zealand public support for cat population control measures and cat management. They recommended that legislation related to this problem should be reviewed [26]. Therefore, this current study aims to investigate the perceptions of urban communities in the Klang Valley on stray and companion animal management in Malaysia by focusing on intervention implementation and regulation enforcement. In this context, four stages of questions are applied. 1) Do people in Malaysia do aware that animals are sentient beings? 2) How do different factors contribute to increased stray animal problems in the country? 3) How can individuals and community members manage stray and companion populations within their locality? 4) How can national policy concerning stray and companion animals be designed for the long term? Variations in respondents’ views regarding stray and companion animal management are expected. Therefore, based on the previous studies conducted locally and internationally, the survey will be ethically analyzed on social acceptability, animal welfare, effectiveness, and legal compliance.

The results of this study are invaluable in assisting the authorities to acknowledge that public understanding in stray and companion animal management in the country is of paramount importance. The collated information will invariably contribute justifiable inputs on how the government could draft a sustainable urban development plan by taking into consideration the co-existence of humans and animals residing in the Greater Kuala Lumpur/Klang Valley area.

## The scenario of stray and companion animal management in Malaysia and the importance of research in regulation implementation

Matters relating to companion and stray animals in the Klang Valley region are regulated based on different municipalities. The current Ministry of Local Government Guidelines emphasizes stray dog animal control, resulting in most captured animals being euthanized [27–30]. There are few federal-based national regulations for protecting companion animals, including dogs and cats, in the region. The Animal Welfare Act 2015 is the only legally binding document that prevents cruel actions toward animals while promoting their welfare [27]. The Act has only been enforced since 2017 despite being passed by the Malaysia Parliament in 2015. Therefore, is a need for in-depth, progressive studies on aspects of its implementation. Implementing national legislation on animal matters is the sole prerogative of the Department of Veterinary Malaysia and municipal authorities. However, commitments toward providing minimum standards for treating and protecting companion animals remain under the purview of the public community.

In 2009, the OIE adopted animal welfare standards applicable to stray dog population control in different countries worldwide to reconcile with the existence of the Terrestrial Animal Health Code. The OIE standards are not immediately binding but represent a fundamental tool to combat zoonotic diseases and other nuisances that stray and free-roaming animals generate. According to these recommendations, once stray dogs are released into the territory, they should be returned to a place that is as near as possible to the place of capture, their welfare should be regularly monitored, and they should be easily identifiable on sight (e.g., visible collar) to avoid unnecessary recapture. It also recommends that if this method is adopted, awareness of the program within the local community should be increased to ensure widespread understanding and support. Article 7.7.7 (4) emphasizes that continuous evaluation should be carried out in particular milestones to check whether the program has the desired and stated impact. Depending on the required objective, the Article suggests several approaches should be adopted (Table 1).

**Table 1:** On-field information gathering approaches

Sources of information for monitoring and evaluation purposes for stray animal population control:	
a) Feedback from the local community	(e.g., through the use of structured questionnaires ^a^, focus groups, or “open format” consultation processes);
b) Records and opinions obtained from relevant professionals	(e.g., veterinarians, medical doctors, law enforcement agencies, educators);
c) Animal-based measurements	(e.g., direct observation surveys of population size and welfare status)

^a^The present study focuses on this approach

Studies conducted internationally suggest that public opinion regarding companion animal management differs according to several demographic variables, namely, knowledge and experience, employment status, gender, and religious beliefs [31–34]. Gates et al. (2019) highlighted opportunities to improve owner compliance through desexing, micro-chipping, and registration of dogs and cats in New Zealand [35]. Opinions towards managing stray cats and dogs vary between pet owners and non-owners. A survey by Rand et al. (2019) reveals various views among the public regarding the lethal and non-lethal practices employed in stray cat management in Australia [36]. Ultimately, they conclude that current local legislation does not reflect public views and should be reviewed.

## Methodology

### Location, duration, and design of the study

**Fig. 1:**
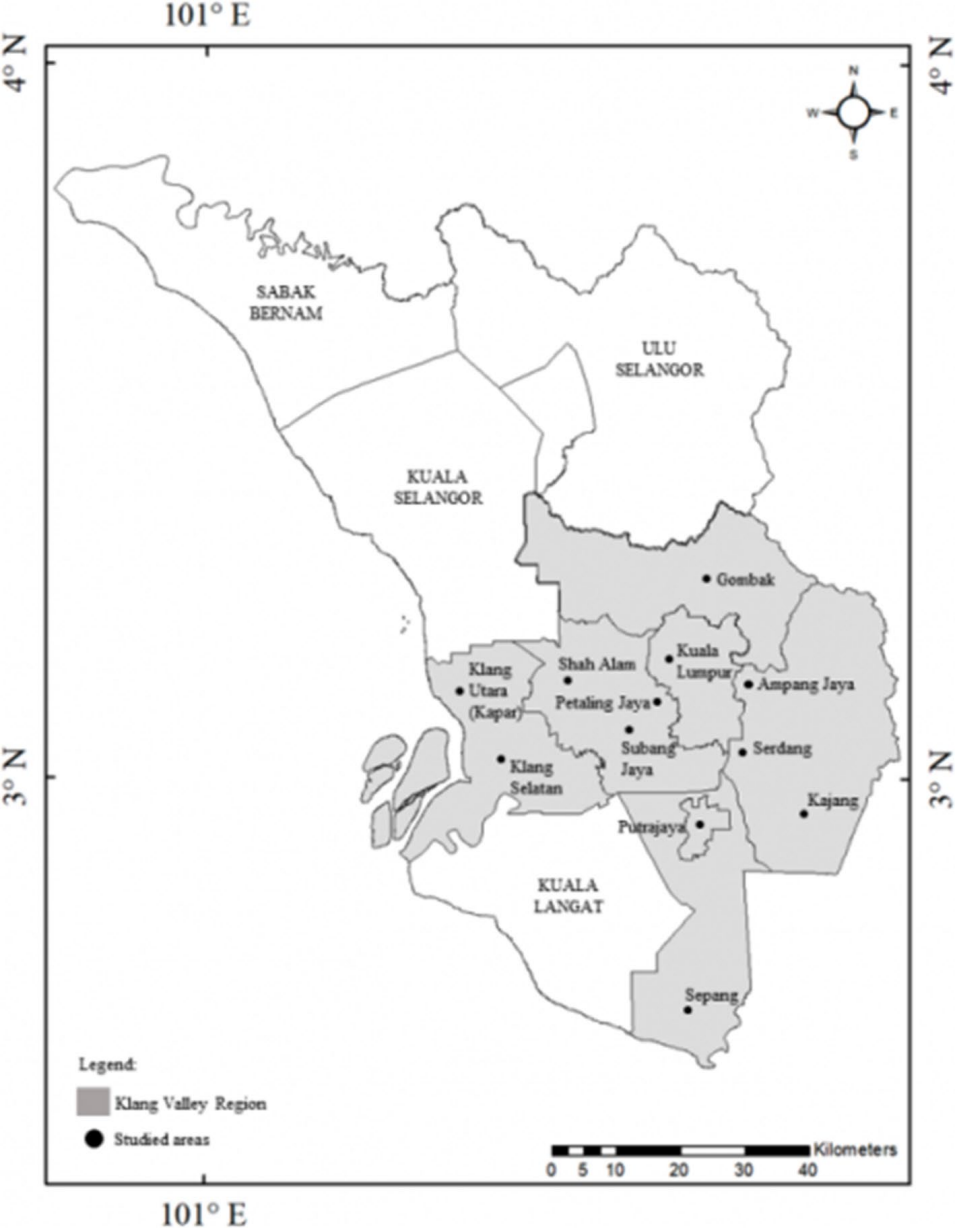
The studies areas within Klang Valley region. Map credit to (Fandi et al., 2020) [39]

The Klang Valley region in Malaysia was chosen as the target location to investigate the perceptions of urban communities on stray and companion animal management in Malaysia. Klang Valley was selected because the region has the highest population in the country, totaling approximately 7,780,000 inhabitants [37]. The sample was randomly selected from among the public (aged 18 years and older) living throughout 10 localities in the Klang Valley (see Fig. 1) map from Fandi et al. (2020) [38], and data collection was undertaken from 1^st^ March 2018 to 31^st^ March 2019. The percentage of approach to all potential respondents are equally distributed to all localities in the Klang Valley, with a higher percentage allocated for Kuala Lumpur due to its larger area size and higher number of population (see Table 2). A simple random sampling technique was used to prevent bias and implement an appropriate methodology that ensured every member of the population had an equal chance of being selected [39].

**Table 2:** Respondents’ demographic shows comparison of number of responses and percentage

Demographic	Demographic Categories	Number of respondents = N	Percentage (%)
	Kuala Lumpur	130	18.5
	Gombak/Selayang/Rawang	62	8.8
	Ampang	62	8.8
	Kajang/Bangi/Semenyih/Hulu Langat	70	9.9
	Petaling Jaya/ Damansara/Kelana Jaya	64	9.1
**Klang Valley area**	Shah Alam	62	8.8
	Subang Jaya/Puchong/Kota Kemuning/Perdana Putra/Saujana Putra	62	8.8
	Klang	60	8.5
	Serdang/Seri Kembangan/Balakong	60	8.5
	Putrajaya/Sepang/Cyberjaya	72	10.2
	Total	704	100.0
	17 – 30 years old	336	47.7
	31 – 40 years old	213	30.3
**Age**	40 – 60 years old	148	21.0
	> above 60 years old	7	1.0
	Total	704	100.0
**Gender**	Male	238	33.8
	Female	466	66.2
	Total	704	100.0
**Religion of faith**	Islam	617	87.6
	Christianity	30	4.3
	Buddhism	30	4.3
	Hinduism	13	1.8
	Sikhism	4	0.6
	Atheism	10	1.4
	Total	704	100.0
**Level of education (Highest)**	PMR/LCE	5	0.7
	SPM/MCE/ O-Level	44	6.3
	STPM/A-Level/ Foundation/Asasi	27	3.8
	Sijil Kemahiran Malaysia (SKM)	4	0.6
	Sijil Teknikal dan Vokasional	3	0.4
	Diploma	126	17.9
	Advanced Diploma	10	1.4
	Bachelor Degree	318	45.2
	MBBS/Dentistry/Veterinary Degree	6	0.9
	Master Degree	140	19.9
	Doctoral Degree	21	3.0
	Total	704	100.0
**Marital status**	Single	359	51.0
	Married	333	47.3
	Divorced	12	1.7
	Total	704	100.0
**Type of current working services**	Government service/ GLC	289	41.1
	Private	182	25.9
	Non-Government Organization (NGO)	8	1.1
	Self-working/Freelancer	29	4.1
	Student	180	25.6
	No working	16	2.3
	Total	704	100.0
**Average household income (per month)**	≤ RM 950.00	68	9.7
	RM 950.01—RM 3, 860.00	258	36.6
	RM 3,860.01—RM 8,319.00	272	38.6
	≥ RM 8,319.01	106	15.1
	Total	704	100.0
**Keeping pet currently**	Yes	299	42.5
	No	405	57.5
	Total	704	100
**Keeping pet previously**	Yes	533	75.7
	No	171	24.3
	Total	704	100

The questionnaire was adopted and modified from previously published work [25, 26, 35, 40]. The questionnaire was designed to apply to all participants, regardless of whether they kept animals. To avoid confusion among respondents about the etymology of companion and stray animals, the animals were defined as follows:

- A companion animal is a common domestic animal, such as a cat or dog, that lives with humans and depends on humans for its welfare.
- A stray animal is a companion animal (e.g., cats and dogs) that are lost or abandoned and lives as an individual or in a group. Stray animals live around the centers of human habitation.

The questionnaire was divided into the following five sections:

- Human and animal continuum of knowledge and awareness
- Causes of the stray animal population
- Management of the stray animal population
- National Strategy on Stray and Companion Animal Management
- Information on respondents’ backgrounds.

In the first section, respondents were asked about their knowledge regarding the continuum of human and animal relationships, including awareness of rights and animal welfare protection. Respondents were asked whether they agree with the following statements: a) Malaysia currently provides viable acts and regulations as guidelines for the protection of animal welfare; b) people adhere to animal based-rules and regulations; c) Animals have a creative mind with the potential to reason; d) Animals possess the capacity to have feelings, such as love and pleasure; e) Animals possess the capacity to have feelings, such as pain and suffering; f) An animal should be given moral consideration equivalent to a human being whilst still alive; g) Animals should be treated equally to a human being, such as being given inheritance rights; h) Animals should be treated equally to a human being, such as being given citizenship rights; i) Dead animal carcasses should be given the same respect as when they were still alive, and j) Religious knowledge is imperative for animal welfare protection. Respondents could select multiple answers in each section.

In the second section, respondents were asked for their opinions regarding the claim stray animal issues in Malaysia cannot be resolved effectively. Choices of possible answers encompassed the following reasons: a lack of general public awareness of animal care, limited promotion through media platforms, the burden of animals’ behavior, financial issues, the ineffectiveness of the existing national strategy to manage stray animal populations, the scarcity of animal registration platforms, lack of enforcement, a limited number of competent enforcement officers, lack of CCTV at hotspot areas, the inadequate number of compounds, pet shops discarding animals, ineffective provision of desexing treatment, religious/cultural sensitivity issues, the ineffective approach of animal rescuers, and the limitations of protection sanctuary area(s) for stray animals. Respondents could select multiple answers in each section.

In the third section, respondents were asked for their opinions on how to manage the stray animal population effectively. The range of techniques includes lethal methods such as shooting and euthanasia or mercy killing. Respondents may choose suspending pregnancy (using non-surgical method of contraception) as a reversible method for blocking fertility or termination of pregnancy as an abortion measure. In relation to the surgical approach, Trap-Neuter-Return (TNR) allows strays to be humanely trapped, neutered or spayed, and then released back into their original habitat. Desexing/sterilization using the neutering method (non-TNR) is commonly applied to control the pet population and reduce unwanted behaviors such as aggression and roaming. Other available options are treatment and vaccination of animals exposed to disease, socializing with public communities, re-homing/ relocation, readopting, and leaving the animals alone. Respondents could select multiple answers in each section.

In the fourth section, respondents were asked for their opinions on the national Strategy of Stray and Companion Animal Management. Respondents chose answers based on the suggestions, which were was: limit the number of household pets, embargo for certain types of high population animal species such as cats and dogs, compulsory animal registration, strengthening the national strategy on animal breeding policies, increase financial grants under the National Companion Animal Welfare Management Program, animal containment in a designated time, no animal roaming without the owner’s supervision, compulsory animal handling training, animal training classes delivered by an animal trainer, animal’s right to inherit wealth from their owners, compulsory animal insurance for pets, accelerating the use of ICT to monitor, track and manage an animal more efficiently, adhering to religious teaching values, incorporating an animal welfare related-course into the mainstream education curriculum, managing animal protection area through the Blue Ocean strategy initiative, establishing more animal welfare ranger posts such as animal police, residential community brigades and stray animal attendees, community service for offenders in animal welfare cases, fines imposed on those who feed stray animals, increasing fines for anyone who abandons/loses any animal (especially in public areas), prohibiting the public feeding of animals in public areas and commercial centers, reducing the selling of cats and dogs in pet shops, exporting numbers of unwanted animals to other countries, and exporting unwanted stray animals to countries that classify domesticated animals such as cats and dogs as food sources. Respondents could select multiple answers in each section.

In the fifth section, respondents were asked to provide demographic characteristics through nine (9) items: gender, age, religion of faith, level of education (highest), marital status, type of working service, average household income, and previous and current pet ownership. To get additional perspectives, current information on keeping animals was also requested, such as types of pets, procedures undergone by each pet, reasons for not undergoing any pet-keeping procedure, and source of pet acquisition. If the respondents had previously owned a pet, they were asked to state what happened to the animal. Respondents were also asked whether they wanted to keep an animal in the future.

### Sample size, data collection, & statistical analysis

This questionnaire was completed face-to-face and online by 704 Malaysian adults (aged 18 years and above) selected using a random sampling technique. The sample size in this current study is based on the calculation method suggested by Daniel (1999) [41]. To calculate the minimum sample size, a confidence level of 95% and a sampling error of 4% were considered:

$$sample size=\frac{\frac{{z}^{2}\cdot p(1-p)}{{e}^{2}}}{1+(\frac{{z}^{2}\cdot p(1-p)}{{e}^{2}N})} =\frac{\frac{{1.96}^{2}\cdot 0.5(1-0.5)}{{0.04}^{2}}}{1+(\frac{{1.96}^{2}\cdot 0.5(1-0.5)}{{0.04}^{2}\mathrm{7,780,000}})}$$

$$\mathrm N=\mathrm{population}\;\mathrm{size}\;\cdot\mathrm e=\mathrm{Margin}\;\mathrm{of}\;\mathrm{error}\;(\mathrm{percentage}\;\mathrm{in}\;\mathrm{decimal}\;\mathrm{form})\;\cdot\mathrm z=\mathrm z-\mathrm{score}$$

Two techniques used in survey data collection were instrumental in ensuring data were collected systematically. First, the survey undertook in public engagement areas such as central shopping areas and public recreational places. A used tab is powered by the mobile survey platform developed by Survey Monkey, which helps provide paperless questionnaire sheets. Alternatively, the traditional printed paper version has been utilized. This technique facilitated data collection face-to-face with the target audience.

Second, the survey was conducted using an online intermediator, Survey Monkey apps. Access to the survey was provided through a web link, email invitation, and social media [42]. Those who volunteered to participate clicked on a link in the message that connected them directly to the survey site. To distribute survey sheets online, participants were recruited using a “virtual snowballing” technique [43]. This involved requesting personal and professional contacts of the research team by email or social media (e.g., Facebook.com or WhatsApp) to complete the survey and then forward this request to their personal and professional contacts. This method is widely used and was chosen to enroll an adequate number of respondents quickly, automatically, flexibly, and in a time-saving manner.

It took approximately 10–15 min for each respondent to complete the survey. Further details about the study such as the information on the direct impacts of the certain procedures to health and welfare of animals were not provided to avoid potential bias in attracting respondents with a greater empathy or interest in animals. Before commencing the survey, the respondents were provided with an information sheet outlining the length of the survey, the anonymity and confidentiality of the information they provided, and their right to withdraw at any time, including up to four months after the completion of the survey. At the end of the survey, respondents were asked to leave their email addresses to be easy to retrace whenever researchers needed to recheck or rechange their answers. However, providing an email address was entirely voluntary.

After completing the survey, respondents were provided with a take-home information sheet which included a unique number identifier they could use should they wish to withdraw their responses at a subsequent date. This research has undergone the necessary process of scrutiny and was approved by the University of Malaya Research Ethics Committee (UMREC) (Ref no. UM.TNC2/UMREC–250) to ensure the integrity of ethical aspects concerning data collection and handling.

All statistical analysis of the sorted data was performed using Microsoft Excel and then exported into the software “IBM Statistical Package for Social Sciences, Statistics 28” (IBM SPSS Statistics 28) [44]. The descriptive statistics analysis enables researchers to present the summaries of the sample, and spatial variation was generated to represent the response rate of survey respondents. A Chi-Square Test for Association was then applied (p < 0.05) to discover if there is a relationship between respondents’ backgrounds and their answers. Only statistically significant results are presented in this paper. Percentages (%) were calculated based on the total number of answers respondents gave to specific questions.

## Results and discussion

### Information on respondents’ backgrounds

**Fig. 2:**
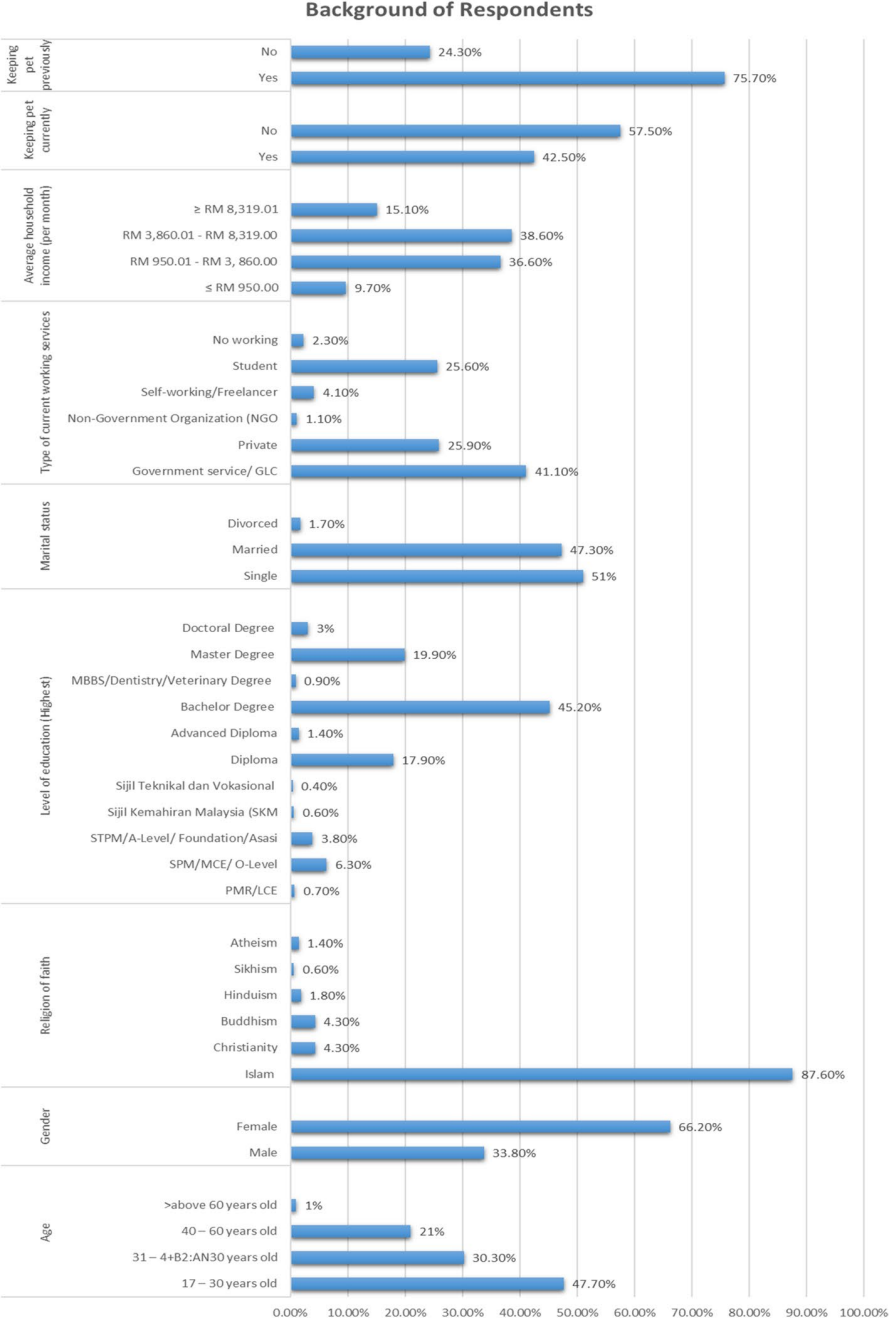
Demographic characteristics of respondents

Based on the data summary in Fig. 2 and details in Table 2, 704 respondents participated in this study, of whom 66.2% were females, and 33.8% were males. The data also indicates that most respondents were between 17 and 30 (47.7%). The majority professed the religion of Islam (87.6%). In terms of marital status, the majority of respondents were single (51.0%), followed by married couples (47.3%). More respondents (38.6%) came from a middle-income family, defined as RM 3,860.01—RM 8,319.00, than a low-income household (36.6%), defined as RM 950.01—RM 3, 860.00. In terms of profession, most respondents were civil servants or employees in government-related agencies or companies (41.1%), followed by students (25.6%) and private-sector workers (25.9%). Regarding education level, most respondents were well-educated, with 45.2% having obtained a bachelor’s degree.

Regarding animal-keeping experience, 42.5% of respondents are keeping pets and 57.5% respondents are currently non-pet keepers. 75.7% of respondents responded yes when asked whether they had previously kept any animal, and 24.3% responded no.

- About current animals

During the period of study, terrestrial domesticated animals, mainly cats, were recorded as the most popular pets (67.6%, 246/364), followed by dogs (8.2%, 30/364) and accumulative of other species of pets (24.2%, 88/364). (see Table S1 Supplementary data for survey).

b) About previous animals

For various reasons, some owners no longer keep their previous animals (see Table 3). A total of 666 is an accumulative response to every single “yes” answer to this question. Most previously owned animals died because of disease (s), unexplained health complications, or illnesses associated with age (33.5%, *n* = 223), followed by those that were lost and unfound (*n* = 175, 26.3%) and those that died because of accidents such as being killed by a vehicle or jumping from high-rise because of a lack of proper surveillance (*n* = 135, 20.3%). Other reasons made up less than 9% of the responses.

**Table 3:** What happened to previous pet animals?

Description	Frequency	Percentage
Dead due to accidents such as was killed by a vehicle or jump off due to lack of proper surveillance	135	20.3%
I give the animal(s) to third parties, such as surrender to an animal center, neighbor, friend, or sell to others	59	8.8%
I discarded the animal(s) to open areas such as residential areas, towns, markets, or recreational zone	23	3.4%
Dead due to disease(s), unexplained health complications, or illnesses associated with age	223	33.5%
Dead due to abuse and violence	12	2%
Been stolen	35	5.2%
Lost and unfound	175	26.3%
Taken by authority	1	0.1%
I left them alone/unattended	3	0.4%
**Total responds** **(say “yes” to every single answer as stated in the list above)**	**666 (100)**	

When asked what happened to their previous animal, the answers given were influenced by several demographic variables, including gender and marital status. Males were less likely than females to respond that their pet died because of an accident (chi-square = 7.619, *p* < 0.01). Counterintuitively, another recent research indicates females are more concern on the issues of welfare of animal than male [45]. However, it is important to note that accidents can happen to anyone who owns a pet and it is not necessarily gender-specific. It is essential to ensure that pets are supervised and kept in a safe environment to prevent accidents from happening. Some common accidents that can result in pet injuries include leaving potentially hazardous items out, such as cleaning products or sharp objects, not supervising pets around other animals, or allowing pets to roam free in areas where they may be at risk of being hit by a car.

Married couples and single people were less likely to respond that their previous animal died because of disease(s) (including died because of various diseases, unexplained health complications, or illnesses associated with age) than divorced people (chi-square = 11.011, *p* < 0.01). Divorced people may risk their pet to disease if one person in the divorce gets the pet and the other doesn't. The pet may experience stress and anxiety from the separation and change in environment. Additionally, if the pet is shared between the two households, it may be exposed to different environments, which can increase the risk of disease.

### Human and animal continuum of knowledge and awareness

Regarding respondents’ knowledge and awareness about animal rights and protection, Table 4 reveals that most respondents believe Malaysia currently provides viable acts and regulations as guidelines for protecting animal welfare (76.1%). Legislation often stipulates only the minimum standards for the protection of animals, and its effectiveness depends on the level of enforcement [46]. For example, Malaysia Animal Act 1953 requires the stray animal population, predominantly dogs, to be controlled, and the essentiality of giving good welfare treatment to an animal in the Animal Welfare Act 2015 [27].

**Table 4:** Respondent’s knowledge and awareness about animal rights and protection

Description	Agree (%)	Disagree (%)
Malaysia currently provides viable acts and regulations as a guideline for the protection of animal welfare and conservation	536 (76.1)	168 (23.9)
People in this country generally adhere to animal welfare rules and regulations	348 (49.4)	356 (50.6)
Animals have creative minds and potentially enabling them to reason	551 (78.3)	153 (21.7)
Animals possess the capacity to have feelings, such as (loving and pleasure)	695 (98.7)	9 (1.3)
Animals possess the capacity to have feelings, such as (pain and suffering)	702 (99.7)	2 (0.3)
An animal should be given moral consideration equivalent to a human being whenever it is alive	348 (49.4)	356 (50.6)
Animals should be treated equally to human beings, such as being given inheritance rights	204 (29.0)	500 (71.0)
Animals should be treated equally to human beings, such as being given citizenship rights	169 (24.0)	535 (76.0)
Dead animal carcasses should be respected, like when they are still alive	675 (95.9)	29 (4.1)
Religious knowledge is imperative for animal welfare protection	675 (95.9)	29 (4.1)

However, achieving the objective of both Acts could be stunted due to implementation factors and public adherents. The survey results indicate that slightly less than half of the respondents agreed that people in Malaysia adhere to animal welfare based-rules and regulations (49.4%). There have been reported 463 animal abuse cases across Malaysia in 2016, followed by a 10 percent increase in 2017, which made the cases reach 510. Subsequently, a 30 percent increase in 2018 became 662 reported cases. Moreover, there was also an increase in animal abuse cases from January to June 2019. More than 90 percent of these cases involved dogs, followed by cats [47].

Most respondents agreed that animals could have feelings, such as love and pleasure (98.7%) and pain and suffering (99.7%). For good sensation feelings, dogs exhibit a pleasurable oxytocin release when being stroked by a human [48]. An animal might eat less food and exhibit unusual behavior characteristics for pain-reflection behaviors. Social behavior is suppressed, agitated, may emit characteristic distress calls, experience respiratory and cardiovascular changes, inflammation, and release of stress hormones [49, 50]. Cross-species empathy also does exist in an animal when it experiences emotional contagion in response to other species’ feelings and emotions [51].

Interestingly, previous studies show cats able to produce food soliciting’ purrs were also perceived as more urgent and less pleasant-sounding by humans. They were also noted to include high-frequency voiced components similar to those produced by human infants when crying [52]. Other studies show that a large percentage of pet owners report consistent signs of jealousy in domestic pets, including horses, birds, and cats. More research into the social emotions of animals other than dogs and primates may reveal that jealousy is more widespread than it appears to be [53].

78.3% of respondents believe that animals have creative minds, potentially enabling them to reason. This discovery corresponds with Jardim-Messeder et al. (2017) study that found that dogs may have about twice the number of neurons in their cerebral cortexes as cats have [54]. Neurons are the basic information processing units; thus, the more units in the brain, the more cognitively capable the animal is, which suggests contributing to the intensity of intelligence level. Previous research also has shown that animals can remember specific events, use tools and solve problems [55]. However, the question of precisely what that means – whether they are making rational decisions or simply reacting to their environment through mindless reflex – remains a matter of scientific dispute and requires further research. Take dogs as an example, we recognize their ability to rear sheep, rescue victims, providing safety and be helpers to humans or their owners. However, one limited study of 18 household dogs found that they lacked spatial memory and were more focused on the “what” of a task rather than the “where” [56]. Another study of canine cognitive abilities found that dogs’ capabilities are no more exceptional than those of other animals, such as horses, chimpanzees, or cats [57].

Regarding animal rights, respondents were not inclined to confer an animal with inheritance rights (71.0%). Likewise, an animal’s citizenship rights were considered inappropriate (76.0%). One of the logical reasons is that animals do not possess the capacity to survive in a man-made world. In many cases, pet owners inherited exceeded amount of wealth from their pets [58]. However, this norm cannot be accepted because accommodating an animal with lavish wealth does not guarantee that the animal will have a wonderful life. In turn, the animal needs good treatment with the capacity of a pet animal (provided by humans). Most benefits from the wealth will not give the animal any meaning, but the wealth executor (a human) will get all the benefits. The pet animal cannot compute a complex calculation concerning inherited wealth, but only humans can do that. Suppose the pet animal has progeny/offspring. In that case, the benefit will apply to them only after the human has managed the wealth as the only trusted wealth executor.

Most respondents (95.9%) from the survey also believe that dead animal carcasses should be respected, like when they are still alive. When a cat or dog dies, the corpse will usually be buried to acknowledge the service given by the animal to its owner. The animal kingdom also observed that adult dogs conceal other dead animals to respect the corpse [59]. Both situations convey that respecting the deceased is part of the instinct of a living being.

Slightly more than half of the respondents were not agreeable (50.6%) to the statement that animals should be given moral consideration equivalent to human beings whenever they are alive. At the same time, almost half of the respondents were agreeable (49.4%) to the statements. In reflection, the results show that many respondents still think, as Regan and Singer (1989) emphasize, that humans are the only creatures with the willpower to take morally permissible actions toward animals [60]. Animals may have the desire, but humans have the autonomy to evaluate their actions rationally [61]. Therefore, how humans can address their moral obligation to animals is essential. In Malaysia, the last tenets of Rukun Negara, “*Kesopanan dan Kesusilaan”* or mutual respect and good social behavior, can be a relevant maxim for human-animal interaction. Although we tend not to give similar consideration to them, our behavior and how we treat them properly as animals showcase the quality of humanity we bear.

### Causes of the stray animal population

Respondents were asked for their opinions on the claim that stray animal issues in Malaysia cannot be resolved effectively (see Table 5). Demographic variables were thought to influence the respondents’ answers.

**Table 5:** Reasons/Opinions that stray animal issues in Malaysia cannot be resolved effectively

Description	Frequency (n)	Percentage (%)
Lack of general public awareness in animal care	495	70.3%
Media platforms seldomly touch on animal welfare as a topic of discussion	311	44.2%
The owner feels burdened with the animal’s behavior	211	30.0%
Lack of financial assistance	303	43.0%
Ineffectiveness of National Strategy in place to manage stray animal populations	322	45.7%
The scarcity of animal registration platform	184	26.1%
Less strict enforcement, particularly for critical cases	290	41.2%
A limited number of competent enforcement officers	197	28.0%
Lack of CCTV at hotspot area	110	15.6%
The amount of compound for animal welfare offenses is inadequate	228	32.4%
Pet shops discard away without killing the unneeded animals	103	14.6%
Veterinary services are ineffective in providing de-sexing treatment	120	17.0%
Religious/cultural sensitivity issues in Malaysia	171	24.3%
The ineffective approach of animal welfare /rescue NGO	147	20.9%
The limitation of stray animal protection sanctuary area(s)	296	42.1%
Stray animal is not an issue in Malaysia. The current situation is fine	18	2.6%

This research indicates that 70.3% of respondents with those who previously had pets were more likely to choose lack of general awareness among the Malaysian public as the reason than the previous non-pet owner (chi-square = 8.588, *p* = 0.003). Stray population contribution factors involve people letting animals roam away without careful monitoring [62], removing/relinquishing unwanted animals to open-area such as housing, market, recreational, or city areas [63] or feeding a stray animal without taking any initiative to take care of it [64]. Non-pet owners are more likely than current pet owners to select the statement as the reason for the unsettled stray animal population in the country (chi-square = 27.171, *p* < 0.001). However, inadequate knowledge of animal care is often thought the owner fails to seek information and treatment for their animal’s health or behavior problems [65].

45.7% of respondents with non-pet owners were more likely than current pet owners to select the ineffectiveness of the National Strategy to manage stray animal populations as a reason (chi-square = 7.387, *p* = 0.007). Moreover, those who previously had pets were more likely to choose it as a reason than previous non-pet owners (chi-square = 5.434, *p* = 0.020). For example, the Perak State Government is still in the process of drawing up an initial plan for the holistic management of abandoned dogs because various dog cases involving them impact community safety [66]. Meanwhile, companion and stray animals matters are currently subordinate to other significant portfolios such as public health service, pollution control, and urban housing planning governed by the municipal authority [67]. In addition, there is no specific secretariat in Malaysia that looks at companion animal matter as a whole concept. Some countries have already initiated it, even though it should be more comprehensive. For example, the Government of South Australia has established Dog and Cat Management Board. The Board takes a leadership role by empowering councils with the tools they need to build safer communities, combatting the problem of increasing dog attacks, encouraging the proactive management of cats, and educating the community about safe behavior around dogs. The Board breaks down council boundaries by facilitating quickly identifying and returning lost pets to their owners [68].

Animal shelters or sanctuaries are believed to provide temporary protection for millions of underprivileged and unowned cats and dogs [69]. However, less than half of the total respondents (42.1%) with non-pet owners are more likely than pet owners to choose the limitation of stray animal protection sanctuary area(s) as the reason (chi-square = 9.062, *p* = 0.003). Also, those who previously had pets were more likely to choose this as a reason than the previous non-pet owner (chi-square = 4.565, *p* = 0.033). There have been growing concerns about how shelters solve or exacerbate the underlying problems of unwanted or overpopulated stray dogs and cats [40] (Protopopova & Gunter, 2017).

Sentiment upon the status of less strict enforcement in the country indicates that less than half (41.2%) of the respondents agreed with non-pet owners more likely than pet owners (chi-square = 4.817, *p* = 0.028). Similarly, those who previously had pets were likelier to choose this reason than previous non-pet owners (chi-square = 4.173, *p* = 0.041). Concerning the status of the Animal Welfare Act 2015, it is considered a brand-new statute. The number of cases that have been charged under the Act so far is relatively very few and in very small proportions.

30.0% of respondents agreed that the owner feels burdened with the animal’s behavior as the cause of the stray population. Although the rate of agreement of this current study is low, a previous study in Melbourne, Australia, indicates reasons for pet relinquishment, including being unable to care for the animals (31%), moving/accommodation issues (27%), and behavior issues (12%) [70]. Aggression is one of the main reasons owners relinquish pet cats and dogs [71–73]. As the relinquished animals need to survive and breed (if not being desexed by the owner), they are likely to gather in urban dumping sites in search of feeding opportunities [74]. Consequently, it increases the threat of zoonotic disease transmission to humans and other animals [75].

This study observed small degrees of respondents’ agreement on the number of competent officers in the country (28.0%), with non-pet owners than pet owners more likely to choose them as the causes for the unsettled stray animal population in the country (chi-square = 5.390, *p* = 0.020). For example, this matter is also being influenced by the emergence of reports of abuse of power and corruption by state enforcement officers [76]. More convincing, 32.4% of the respondents also agree that fines for animal welfare offenses are inadequate. The severity of penalties for cruelty toward animals historically has been very mild, and countless complaints lodged to the authorities were unheeded even when the perpetrators were identified [77].

### Management of stray animal population

Respondents were asked to give their opinion on adequately managing the stray animal population (see Table 6). Demographic variables influenced these answers.

**Table 6:** Respondents’ opinions on adequately managing the stray animal population

Description	Frequency (n)	Percentage (%)
Treatment and vaccination of animals exposed to the disease	409	58.1%
Readopting	282	40.1%
Desexing/Sterilization using neutering method (non-TNR).”	251	35.7%
Re-homing/ Relocation	240	34.1%
Trap-Neuter-Return (TNR)	237	33.7%
Socializing with public communities	95	13.5%
Suspending pregnancy (using non-surgical method of contraception)	75	10.7%
Lethal methods such as euthanasia or mercy killing	66	9.4%
Do nothing. Leave the animals alone	54	7.7%
Termination of pregnancy (planned abortion)	32	4.5%
Lethal methods such as shooting	13	1.8%

The current study indicates that more than half of the respondents, or 58%, support effectively treating and vaccinating animals exposed to disease to manage the stray animal population. With proper vaccination, many non-vaccinated animals can achieve ample immunity against opportunistic pathogens, which could cause detrimental health effects later in their lives. Since 2015 Malaysia has lost its status as a free-rabies country. The Malaysian government has promoted pet mass vaccination, especially in disease-outbreak states [78]. Nevertheless, other than the requirement for rabies vaccination in outbreak locations, general cat and dog vaccinations have not been made compulsory, making pet owners less likely to support this measure than the non-pet owner (chi-square = 18.335, *p* < 0.001). Also, gender attribution may explain why more female than male respondents choose vaccination as the preferred disease prevention method (chi-square = 6.080, *p* = 0.014). It is in line with many previous findings reported that females tend to express more positive attitudes toward individual animals compared to male people [79–81]. The benefits of vaccinations and other antiparasitic treatments, when combined with desexing or sterilization, offer a significant improvement in public health by reducing the stray dogs’ population and spread of rabies infections happened [82–89].

The results indicate that 40.1% of the respondent’s selected readoption as a solution. Although the agreement rate is less than half of the total respondents, the rate is considerably much better than a study in Penang which found that only 19% of respondents believe readopting can effectively reduce the stray animal population [25]. Once adopted, an animal risks being returned to the animal shelter due to behavioral problems [90]. Thus, new owners and adopters would suggest considering the risk and benefits of adopting any stray animal and properly planning specific interventions before, during, and after adoption. These include in-house safety and animal behavioral assessment, pet identification devices, pet care educational materials for owners, training classes, subsidized veterinary health services, and emergency assistant responses. A long-term engagement program with prospective owners will potentially contribute to higher adoption success, and a lesser risk of relinquishment as prospective owners will make informed decisions when selecting suitable animals to adopt [40].

Overall, 35.7% of respondents agreed that respondents who previously had pets were also more likely to support desexing/sterilization using neutering method (non-TNR) initiatives than those who had never been animal owners (chi-square = 6.568, *p* = 0.010). The Terrestrial Code Article 7.7.6 World Organisation for Animal Health (OIE) (2016) has recommended reproductive control as a combined measure alongside conventional culling for dog population control [91]. At the same time, previous research in Penang showcases that only 14% of respondents agree with desexing or neutering as an effective measure in reducing the stray animal population [25]. The support rate is considerably low as there is a lack of measurable results on the effectiveness of neutering in Malaysia for the long run 5 -10 years. Dog reproductive control is currently more socially acceptable than culling in countries such as Italy [92], India [86, 89, 93], and Brazil [94].

In addition, 34. 1% of respondents were found to agree with re-homing and relocation, with the respondent with pets less likely to support this measure than the non-pet owner (chi-square = 28.060, *p* < 0.001). It is parallel with the Penang study, which revealed that 31%, the majority, favored providing a good animal shelter scheme that would reduce the stray animal population [25]. Stray protection areas, shelters, halfway homes, or sanctuaries could also be ideal spaces to retrain animals with unfavourable behaviors. There are include as high temperament levels and non-hygienic characteristics for a proportion of stray cats and dogs before they can be set free in their forever home via a readopting program [95].

Notably, 33.7% of respondents agreed that people who previously had pets were more likely to support Trap-Neuter-Return (TNR) initiatives than those who had never been an animal owner (chi-square = 8.382, *p* = 0.004). In the Penang study, 31% of respondents believed the TNR scheme could reduce the stray animal population effectively. There was a similar level of agreement (31%) for the Trap & Euthanize (TE) scheme. However, only 7% of respondents preferred Trap Neuter as a viable approach to reducing the stray animal population. The authors believed this happens because respondents want a positive and humane approach to the Free-Roaming Dogs (FRD) issue rather than TNR [25]. TNR is well-known as a non-lethal method that alters an animal’s sexual organs so it can no longer reproduce [96].

However, the status of vaccinated, neutered, and microchipped of Trap-Neuter-Release (TNR) dogs and cats can be the source of contentiousness for many residents in the community where the program took place. People may be concerned about public safety, zoonoses spread, and other nuisances caused by free-roaming animals. It is worthy to note here that any person who conducts TNR on animals without providing adequate protection to the animal may risk infringing Sect. 33(2), of the Animal Welfare Act 2015. It happens if they allow an animal to be oppressed, fail, or neglect them to have a shelter, and abandon them till they are likely to suffer trauma, pain, or suffering due to relocation, starvation, thirst, injury, or illness. Above all, TNR effectiveness depends on the extent to which mass migration of animals from outside of the colony can be prevented (such as controlling urban waste), high levels of desexing of individual animals, high rates of adoption, and the allowance of time for natural attrition to occur [26].

Other stray animal population reduction measures that received a minimal level of agreement by survey respondents were socializing with public communities (13.5%). The choice of suspending pregnancy (using non-surgical method of contraception) (10.7%) include hormonal treatments, such as progestins, androgens, or analogs of gonadotropin releasing hormone (GnRH) [97]. Termination of pregnancy (planned abortion) (4.5%) measures in both dogs and cats include administration of prostaglandin F2alpha, dexamethasone, combination drug protocols or estrogens.

Concerning lethal methods, shooting elicited the lowest respondent acceptance (1.8%) due to its apparent brutalism in exterminating living beings. Support for lethal methods (e.g., shooting) is very low (1.8%) despite their potential for money-saving, with male respondents more supportive than females (chi-square = 15.279, *p* =  < 0.001), and pet owners were less likely than non-pet owners to support it (chi-square = 6.557, *p* = 0.010). In comparison, sentiment on euthanasia or mercy killing received slightly higher or 9.4% agreement with female respondents more supportive than the male of the procedure (chi-square = 8.534, *p* = 0.003), pet owners are less likely than non-pet owners to support the measure (chi-square = 5.581, *p* = 0.018). It corresponds with the previous study showcasing that 70% of the public disagreed with the Trap & Euthanasia (TE) scheme because it is inhumane to kill animals in general. Only 12% of respondents favored the TE scheme and considered it the most effective solution to stray animal problems [25]. This research also found that males are more likely to support using lethal methods than females. Overall, this current study indicates that male, non-pet owners tend to believe that the euthanasia of stray animals, especially urban strays that potentially spread disease, is humane and significantly more likely to prefer lethal control [36]. By contrast, females were less likely to choose lethal control and were more concerned about animal welfare and rights than males [79].

### National strategy on stray and companion animal management

Respondents were asked to give their opinion on the National Strategy of Strays and Companion Animal Management (see Table 7). Several demographic variables influenced the answers given.

**Table 7:** Respondents’ opinions about the strategy for stray and companion animal management on the national level

Description	Frequency (n)	Percentage (%)
Limit the number of the household pet at any one time	285	40.5%
Executing an embargo (temporary banning) order to prevent a specific type of high-population animal species (such as cats and dogs) from entering (imported) into this country	153	21.7%
Compulsory animal registration to pet’s owner, seller, and breeder	334	47.4%
Strengthen the national strategy regarding companion animal breeding policies	228	32.4%
Increase the financial allocation for funds or grants under National Companion Animal Welfare Management Program	279	39.6%
Animals should be confined to their owner’s property at a designated time, thus less movement and less headache	121	17.2%
No animal roaming without the owner’s supervision	253	35.9%
Periodical animal handling training is compulsory for everyone who is engaged with animal beings	192	27.3%
An animal shall be trained through a special training class taught by an animal trainer	97	13.8%
Animals should be conferred with better rights even on wealth inheritance from their owners	22	3.1%
Compulsory pet animal insurance, where the coverage benefits are extended to its owner too	63	8.9%
Accelerating modernized technologies or ICT devices that are useful for monitoring, tracking, and managing an animal more efficiently	201	28.6%
Acclimatizing to more vital religious teaching values on animal welfare to every individual	219	31.1%
Incorporating animal welfare related-course in the mainstream education curriculum in schools and higher learning institutions	202	28.7%
Encouraging development and management of animal protection areas (such as stray animal sanctuaries and recreational, integrated animal breeding centers, animal halfway home, animal-hostel (pet’s boarding), pet-sitter, and animal-friendly café) through the Blue Ocean strategy initiative based on the cooperative partnership between government, private and public funds	341	48.4%
Establishing more animal welfare rangers posts such as animal police, residential community brigades, and stray animal attendees that are dedicated to patrolling, investigating, and curbing animal cruelty cases throughout the country	236	33.5%
The penalty for community services to offenders involving animal welfare cases	286	40.6%
Fine to whom feeding the stray animal without the intention to keep them as its pet	36	5.1%
Increase the fine for anyone who throws away/loses any animal, especially in public areas	239	33.9%
Pasting more warning notices/messages prohibits feeding animals in public passages areas and commercial centers	45	6.4%
Reducing the selling number of highly populated invasive species of animals in pet shops (including cats and dogs)	107	15.2%
Exporting unwanted animals to a country with minimal numbers of companion animals	3	0.4%
Exporting unwanted stray animals to a country that defines domesticated animals such as cats and dogs as their food sources	0	0%

Suggestions for the creation of a stray animal protection area or sanctuary through public, private, and community integration or a blue ocean strategic partnership achieved 48.4% agreement with those who previously had pets more likely to support the measure than previous non-pet owners (chi-square = 4.451, *p* = 0.035). As the animal sanctuary does not mean a permanent place for cats and dogs to live permanently, the current pet owners were less likely to support the initiative than non-pet owners (chi-square = 5.089, *p* = 0.024) is noted. Therefore, the animal sanctuary should be developed based on sustainable development goals that ensure ecological continuity between animal and human relations. In this sense, it should attract active public involvement by being a recreation place to promote mutual ethnic volunteerism and green entrepreneurship among local citizens. More importantly, it is to promote adoptions via long-term engagement with the fosterer to minimize unnecessary in-house congestion due to over-receiving.

This current study indicates that less than half of the total respondents, or 40.5% of respondents, agree to limit the number of pets per household. The finding parallels other studies indicating, for instance, that 70% of people in New Zealand agree with a proposed limit of two cats per household [26]. This intervention can sound restrictive for some, with pet owners less likely to support it than non-pet owners (chi-square = 44.705, *p* < 0.001). However, an excessive number of pets contribute to the long-standing overpopulation problems by adding some strays in a locality. Conversely, the owner having fewer pets should also reduce the nuisance to other residential communities and encourage owners to give more careful attention to their pets. In this sense, the move by the local authority of Boroondara City, Victoria, Australia, to restrict the number of domestic pets can serve as an exemplary implementation model in Malaysia. Suppose the owner needs to keep more than the permitted number of animals, mammals, or birds on their property for whatever reason. In that case, they must apply for an Excess Animal Permit [98].

47.4% of respondents favor the government imposing compulsory animal registration for pet owners, sellers, and breeders. In contrast, more than 70% of people in an Australian study favored a compulsory dog registration scheme [99]. This requirement is not limited to specific dog breeds but involves cats and other domestic animals [100]. Locally, however, Malaysian regulation only demands dog owners to register their pets, except for cat owners. In reflection of the current study, pet owners were observed less to support the measure than non-pet owners (chi-square = 26.723, *p* < 0.001). Only recently, the Malaysian government, via DVS, started to enforce a brand new MyAnimal Welfare, but this pet registration platform is still in a rudimentary phase. Initially, it only focuses on a few sectors, namely activities regarding animal hostels, animal riding, and herding, animal shelter including stray animal protection, and pet shop and pet trading [101].

Unwanted pets are often given away for logistical or financial reasons. Evidence suggests that pre-surgical euthanasia on pet dogs may be primarily economically motivated simply because treatment costs are too high [102]. In relation, 39.6% of respondents agreed to increase the financial allocation for funds or grants under the National Companion Animal Welfare Management Program. Respondents that previously had a pet were more likely to support an increase in the financial allocation for the fund or grants under the National Companion Animal Welfare Management Program than those who had never been an animal owner (chi-square = 16.737, *p* < 0.001). On the other hand, authorities can be self-sufficient, which has been practiced in South Australia. The authority’s operation is almost entirely funded by a percentage of dog registration fees remitted by councils and breeder registration fees. Dogs and Cats Online, an online database and registration platform for dogs and cats’ ownership related activities, continues to provide savings to councils through registration renewal notices, reduced postage and administration costs, easier annual reporting processes, and streamlined registration. Most importantly, Dogs and Cats Online continues to deliver efficiencies to Authorised Officers to guide and assist councils in establishing cat and dog by laws [103].

Concerning not allowing animals to roam without the owner's supervision, there was 35.9% agreement with pet owners less likely to support the measure than non-pet owners (chi-square = 19.032, *p* < 0.001). Pet containment can actually be beneficial for both pets and their owners. By keeping pet in a safe and secure environment, pet owner can have peace of mind knowing that the pet is not wandering off, getting lost or hurt. This alleviates pet owners’ feelings of sadness or anxiety worrying about their pet's safety. Additionally, pet containment can also prevent home furniture and property damage, which is a great stress relief to pet owners. Overall, pet containment can provide a sense of security and comfort for both pets and their owners [104]. However, this research observed low levels (17.2%) of agreement regarding pet confinement at a designated time. It is somewhat lower than other researchers who reported 36% public agreement toward the measure [26]. However, in other studies, the percentage was lower [105, 106]. Interestingly, the range of agreement was between 45–54% if this measure took place during the night [107]. In Australia, the level of compliance relating to the confinement of cats at night has been reported to vary between 32–80% [33, 110]. Conversely, other research indicates that confinement only at night minimally reduces predation behavior as companion cats primarily hunt during the day [108]. Regardless of whether it takes place during the day or night, the benefits of pet confinement have been documented to control public nuisance caused by unrestrained pets [32, 33, 109]. It reduces the injury rates associated with road accidents and minimizes the risks of encountering other cat and dog attacks [33, 110].

In addition, 33.9% of respondents agreed to increase fines for anyone who discards/loses any animal, especially in public areas. Furthermore, 40.6% of respondents agreed that a community services penalty should be applied to offenders involving animal welfare cases. Both types of penalties could teach the offender not to engage in similar wrongdoings in the future. However, community services are a pragmatic type of punishment driven by a value-laden approach that promotes direct social engagement between offenders and public citizens.

Overall, 32.4% of respondents are in favour of the effort to strengthen the national strategy regarding companion animal breeding policies. However, pet owners are less likely to support the measure than non-pet owners (chi-square = 7.525, *p* = 0.006). Therefore, considering people socio-economic factor (such as pet owner financial ability) is essential while conducting feasibility study for policy implementation. For example, the number of domestic animals sterilized may be increased by offering subsidized sterilization programs to low-income pet owners [111]. This is because the program is accessible to poor and needy caretakers (B40 group members) who need help transporting their cats and dogs to the place where the surgery is performed and back home again. Other options to address this issue include providing services through a network of private veterinary clinics (if enough clinics participate) and mobile surgical facilities.

The survey results also indicate that 33.5% of public respondents agreed with establishing more animal welfare rangers’ posts such as animal police, residential community brigades, and stray animal attendees dedicated to patrolling, investigating, and curbing animal cruelty cases throughout the country.

Furthermore, 28.6% of respondents agreed that those who previously had a pet were more likely to support using modernized technologies or ICT devices to monitor, track, and manage animals more efficiently than previous non-pet owners (chi-square = 6.225, *p* = 0.013). Presenting a physical pet identification tag with QR barcodes would allow anyone to obtain the contact information of pet owners simply by using a smartphone application. Implanting a recognizable pet microchip would allow the authority to access the pet and its owner’s status in detail. Consequently, lost pets are more likely to be reclaimed as pet identity information is continually updated and corrected [112–114]. However, the government must assess and study the risk associated with personal data breaches before implementing the measure.

Concerning education, 27.3% of respondents with pet owners less likely to support the measure than non-pet owners (chi-square = 25.586, *p* < 0.001) agreed that periodical animal handling training should be compulsory for everyone who engages with animal beings. In Duxbury et al.’s (2003) research, the owners of puppies that attended socialization classes run by the shelter from which they were adopted obtained some benefits [115]. Pets were relinquished less frequently than owners that attended socialization classes elsewhere or did not attend any classes [115]. In the current research, 31.1% of respondents agreed with acclimatizing to more vital religious teaching values on animal welfare for every individual. Because many Malaysians are Malay Muslims, reform toward holistic-based education can be translated practically as a residential mosque can be transformed into an animal-friendly mosque. Additionally, 28.7% of respondents agreed with incorporating animal welfare-related courses into mainstream education curricula in schools and higher learning institutions. It is due to the lack of mandatory curriculum subjects that give special attention to the welfare of domesticated companion animals living in human habitats.

Regarding animal education, 13.8% of respondents believed that animals should be trained through special training classes taught by an animal trainer. It aligns with previous research, showing that dogs housed in animal shelters can learn new behaviors, inhibiting problem behavior [116, 117]. It can provide benefits to the prospective owner in making the animal much easier to handle and thus minimizing the risk of relinquishment. However, the suggestion that animals should be trained through a particular training class taught by an animal trainer causes the current pet owners less likely to support the measure than non-pet owners (chi-square = 8.523, *p* = 0.004). Hence, promoting compulsory periodical pet obedience classes should focus on dangerous dog breeds owned by special permits.

Only a small rate, 21.7% of respondents with pet owners less likely to support the measure than non-pet owners (chi-square = 12.314, *p* < 0.001) agreed on embargo (temporary banning) order to prevent a specific type of high population animal species (such as cats and dogs) from entering (being imported) into this country. In addition, 15.2% of respondents agreed on reducing the number of highly populated invasive species of animals sold in pet shops (such as domesticated cats and dogs). However, the impact of applying both measures should be studied in detail, as it may risk the economic balance of trading activities involving companion animals. Alternatively, extensive promotion should be focused on encouraging the public to become adopter families for unowned stray cats and dogs can provide long-term welfare and reduce their population on the streets.

There were minimal agreements on measures such as compulsory pet animal insurance, which 8.9% of respondents agreed on when the coverage benefits also extend to owners. Overall, 6.4% of respondents agreed to post more warning notices/messages that prohibit the public feeding of animals in public areas and commercial centres. There was a 5.1% agreement with the imposition of fines on those who feed stray animals without intending to keep them as pets. Regarding other measures, just 3.1% agreed that animals should be conferred with better rights, such as wealth inheritance from their owners. Only 0.4% agreed to export unwanted animals to a country with a minimal number of companion animals.

No respondent agreed on exporting unwanted stray animals to a country that defines domesticated animals such as cats and dogs as food sources. It is because in Malaysian culture, eating those animals is considered taboo, and any activities related to this, including trading, will attract serious remonstrations from the public.

## Limitations

It is essential to point out that although a standardized sampling method has been utilized, researchers for this study had no control over respondents who independently chose to approach and participate. Consequently, the representative nature of these findings to the people, especially those living in Klang Valley, must be considered cautiously. For instance, most respondents were well-educated, and those who completed the research were above 18. It is due to Klang Valley houses many educational institutions, and people in the area have attended training and classes to live and gain knowledge for their self-promotion and value-added. Male respondents were much fewer than female respondents, who are less than half or about 33%, due to the acceptance rate to joining the survey differed between gender. During survey collection, participants from Malay and Muslim are more likely to accept survey invitations than other races. In this sense, Malay and Muslim perspective factors will dominantly influence the survey result.

Regarding pet-keeping experiences, there is the possibility that respondents who are currently keeping pets might have owned other animals previously. Regarding the type of pets, some owners may have one or more different types of pets at one time. There are also many situations, such as in Table 4, Table 5, Table 6, and Table 7, whereby the total number of respondents exceeds the actual number of respondents. It happens because the respondents may have multiple opinions/answers.

## Conclusion

To conclude, the results of this study indicate that the Malaysian public has showcased their interest in effectively managing the stray animal population. They also favor contributing realistic suggestions to Companion Animal Management that can be utilized as a part of the national strategy to reduce problems associated with strays. Nevertheless, no single solution can provide complete prevention and protection for stray and companion animal management. A successful full-spectrum management scheme requires multiple techniques, measures, and cooperation from the government, owners, and the public.

In particular, respondents that had previously kept an animal expressed increased concern regarding the cause of the stray animal population. Currently, pet owners expressed fewer agreements than non-pet owners regarding the many causes that contribute to stray population problems and suggested improvement strategies. It implies that the willingness of current pet owners to comply with many indicative measures remains low. Thus, additional promotions and education are required to establish an integrated strays and companion animal management strategy. It is recommended that the information gathered from this study should serve as a “door opener” to more detailed studies or surveys of pet-keeping activities, especially those related to the issues of animal welfare, urban ecology, and sustainability.

## Declaration

### Supplementary Information

**Additional file 1.** 

## Data Availability

All relevant data are within the manuscript.
