# Supplementary material for: Public perspectives on strays and companion animal management in Malaysia
Source: BMC Public Health. 2023 Jul 26;23:1428. doi: 10.1186/s12889-023-16276-5 (PMC10373340; doi:10.1186/s12889-023-16276-5)
Supplement: Supplementary file 1 — Additional file 1. [file 12889_2023_16276_MOESM1_ESM.docx]

# Appendix: Supplementary data for the survey

Public Perspectives on Strays and Companion Animal Management in Malaysia

**Table S1: Type of companion animals or pets and frequency reported by survey respondents.**

| **Type of pet** | **Number of responses = N and**  **Frequency (%)** |
| --- | --- |
| Cat | 246 (67.6) |
| Dog | 30 (8.2) |
| **Other animals** |  |
| 1. Fish | 35 (10.0) |
| 1. Aviary | 9 (2.4) |
| 1. Tortoise | 10 (2.7) |
| 1. Rabbit | 7 (1.9) |
| 1. Sugar glider | 3 (0.8) |
| 1. Frog | 3 (0.8) |
| 1. Lizard | 3 (0.8) |
| 1. Snake | 3 (0.8) |
| 1. Crocodile | 2 (0.5) |
| 1. Hamster | 3 (0.8) |
| 1. Goat | 3 (0.8) |
| 1. Hedgehog | 2 (0.5) |
| 1. Macaque | 2 (0.5) |
| 1. Porcupine | 1 (0.3) |
| 1. Insect 2. Civet | 1 (0.3)  1 (0.3) |
| **TOTAL** | **364 (100)** |

## Table S2: Association between respondents' background and answers on what happened to their previous pet animals.

| **Background of Respondents** | **What happened to previous pet animals?** | | | |
| --- | --- | --- | --- | --- |
|  | **Pet died due to an accident** | | **Pet died of diseases** | |
|  | No | Yes | No | Yes |
| Male | 206 | 32 |  |  |
| Female | 363 | 103 |  |  |
| Single |  |  | 244 | 115 |
| Married |  |  | 234 | 99 |
| Divorced |  |  | 3 | 9 |
| Test | | | | |
| Chi squared | 7.619 | | 11.011 | |
| *p*-value | **0.006** | | **0.004** | |

## Table S3: The association between respondents' background (concerning their pet-keeping status) and their opinion about the causes of stray animal populations cannot be resolved effectively.

| **Background of Respondents** | **Causes of stray animal population** | | | | | | | | | |
| --- | --- | --- | --- | --- | --- | --- | --- | --- | --- | --- |
|  | **Lack of general awareness concerning animal care among the Malaysians** | | **Ineffectiveness of a national strategy to manage stray animal populations** | | **Less strict enforcement** | | **A limited number of competent enforcement officers** | | **Limitation of stray animal protection sanctuary area(s)** | |
|  | No | Yes | No | Yes | No | Yes | No | Yes | No | Yes |
| Currently, yes | 120 | 179 | 180 | 119 | 190 | 109 | 229 | 70 | 192 | 106 |
| Currently, no | 89 | 316 | 202 | 203 | 224 | 181 | 278 | 127 | 215 | 190 |
| Test | | | | | | | | | | |
| Chi squared | 27.171 | | 7.387 | | 4.817 | | 5.390 | | 9.062 | |
| *p*-value | **<0.001** | | **0.007** | | **0.028** | | **0.020** | | **0.003** | |
| Previously, yes | 143 | 390 | 276 | 257 | 302 | 231 | 375 | 158 | 296 | 236 |
| Previously, no | 66 | 105 | 106 | 65 | 112 | 59 | 132 | 39 | 111 | 60 |
| Test | | | | | | | | | | |
| Chi squared | 8.588 | | 5.434 | | 4.173 | | 3.003 | | 4.565 | |
| *p*-value | **0.003** | | **0.020** | | **0.041** | | 0.083 | | **0.033** | |

## Table S4: Association between respondents' background and opinions on properly managing stray animals.

| **Background of Respondents** | **Management of stray animal population** | | | | | | | | | | | |
| --- | --- | --- | --- | --- | --- | --- | --- | --- | --- | --- | --- | --- |
|  | **Lethal methods (e.g., shooting)** | | **Lethal methods (e.g., euthanasia or mercy killing)** | | **Trap-Neuter-Return (TNR)** | | **Animal de-sexing/ sterilization (neutering)** | | **Treatment and vaccination** | | **Rehoming/ relocation** | |
|  | No | Yes | No | Yes | No | Yes | No | Yes | No | Yes | No | Yes |
| Male | 227 | 11 | 205 | 33 |  |  |  |  | 115 | 123 |  |  |
| Female | 464 | 2 | 433 | 33 |  |  |  |  | 180 | 286 |  |  |
| Test | | | | | | | | | | | | |
| Chi-squared | 15.279 | | 8.534 | |  | |  | | 6.080 | |  | |
| *p*-value | **<0.001** | | **0.003** | |  | |  | | **0.014** | |  | |
| Currently, yes | 298 | 1 | 280 | 19 |  |  |  |  | 153 | 146 | 230 | 69 |
| Currently, no | 393 | 12 | 358 | 47 |  |  |  |  | 142 | 263 | 234 | 171 |
| Test | | | | | | | | | | | | |
| Chi squared | 6.557 | | 5.581 | |  | |  | | 18.335 | | 28.060 | |
| *p*-value | **0.010** | | **0.018** | |  | |  | | **<.001** | | **<0.001** | |
| Previously, yes |  |  |  |  | 338 | 195 | 329 | 204 | 216 | 317 |  |  |
| Previously, no |  |  |  |  | 129 | 42 | 124 | 47 | 79 | 92 |  |  |
| Test | | | | | | | | | | | | |
| Chi-squared |  | |  | | 8.382 | | 6.568 | | 1.712 | |  | |
| *p*-value |  | |  | | **0.004** | | **0.010** | | 0.191 | |  | |

## Table S5: Association between respondents' background and opinion on National strategy on Companion Animal Welfare Management.

| **Background of Respondents** | **National strategy on Companion Animal Welfare Management** | | | | | | | | | | | | | | | | | | | |
| --- | --- | --- | --- | --- | --- | --- | --- | --- | --- | --- | --- | --- | --- | --- | --- | --- | --- | --- | --- | --- |
|  | **Limit no. of animals** | | **Import embargo on certain animals** | | **Compulsory registration** | | **Strengthen breeding policies** | | **Increase government funding** | | **Free-roaming prohibition** | | **Training for animal handler/ owner** | | **Training for animal** | | **ICT for animal welfare** | | **Animal sanctuary** | |
|  | No | Yes | No | Yes | No | Yes | No | Yes | No | Yes | No | Yes | No | Yes | No | Yes | No | Yes | No | Yes |
| Currently, yes | 221 | 78 | 253 | 46 | 191 | 108 | 219 | 80 |  |  | 219 | 80 | 247 | 52 | 271 | 28 |  |  | 168 | 131 |
| Currently, no | 198 | 207 | 298 | 107 | 179 | 226 | 257 | 148 |  |  | 232 | 173 | 265 | 140 | 336 | 69 |  |  | 195 | 210 |
| Test | | | | | | | | | | | | | | | | | | | | |
| Chi squared | 44.705 | | 12.314 | | 26.723 | | 7.525 | |  | | 19.032 | | 25.586 | | 8.523 | |  | | 4.451 | |
| Fisher exact test |  | |  | |  | |  | |  | |  | |  | |  | |  | |  | |
| *p*-value | **<0.001** | | **<0.001** | | **<0.001** | | **0.006** | |  | | **<0.001** | | **<0.001** | | **0.004** | |  | | **0.035** | |
| Previously, yes |  |  |  |  |  |  |  |  | 299 | 234 |  |  |  |  |  |  | 368 | 165 | 262 | 271 |
| Previously, no |  |  |  |  |  |  |  |  | 126 | 45 |  |  |  |  |  |  | 135 | 36 | 101 | 70 |
| Test | | | | | | | | | | | | | | | | | | | | |
| Chi squared |  | |  | |  | |  | | 16.737 | |  | |  | |  | | 6.225 | | 5.089 | |
| Fisher exact test |  | |  | |  | |  | |  | |  | |  | |  | |  | |  | |
| *p*-value |  | |  | |  | |  | | **<.001** | |  | |  | |  | | **0.013** | | **0.024** | |

## Table S6: Association between respondents' background (concerning their pet-keeping status) and the likelihood of keeping any animal.

| **Background of Respondents** | **Likelihood of keeping any animal in the future** | | |
| --- | --- | --- | --- |
|  | **I cannot decide it for now** | **Perhaps, in the future**  **I would like to keep an animal** | **Absolutely not!** |
| Currently, yes | 181 | 105 | 13 |
| Currently, no | 15 | 255 | 135 |
| Test | | | |
| Chi squared | 294.373 | | |
| *p*-value | **<.001** | | |
| Previously, yes | 181 | 289 | 63 |
| Previously, no | 15 | 71 | 85 |
| Test | | | |
| Chi squared | 121.985 | | |
| *p*-value | **<.001** | | |
